# Pharmacokinetic determinants, clinical efficacy, and safety of teicoplanin in adults: a correlative analysis of plasma concentration and treatment outcomes

**DOI:** 10.3389/fphar.2026.1838465

**Published:** 2026-10-28

**Authors:** Ying-Chao Ma, Yong-Jing Wang, Xia Wu, Zhi-Qing Zhang, Xiu-Ling Yang, Jian-Jun Gu

**Affiliations:** 1 Department of Pharmacy, The Second Hospital of Hebei Medical University, Shijiazhuang, China; 2 Department of Cardiac Surgery, The Second Hospital of Hebei Medical University, Shijiazhuang, China

**Keywords:** adult bacterial infection, adverse reactions, clinical efficacy, influencing factors, plasma concentration, teicoplanin

## Abstract

**Objective:**

The aim of this study was to develop and validate a high-performance liquid chromatography (HPLC) method for the determination of plasma teicoplanin concentrations, identify factors influencing teicoplanin exposure in adult patients, and assess its clinical efficacy and safety.

**Methods:**

A cohort of 125 adult patients who received teicoplanin therapy at the Second Hospital of Hebei Medical University between June 2022 and March 2023 were retrospectively enrolled. Trough plasma teicoplanin concentrations were determined using a validated HPLC method with quetiapine fumarate as the internal standard. Clinical data, including demographic characteristics, infection type, laboratory parameters, and treatment outcomes, were collected. Logistic regression analyses was conducted to evaluate the clinical efficacy of teicoplanin, attainment of target trough concentrations, and factors associated with these outcomes.

**Results:**

The HPLC method demonstrated high specificity, satisfactory linearity over the concentration range of 1.5625–100 μg/mL, and acceptable precision, recovery, and stability. Under the standard dosing regimen, only 45.6% of patients achieved a teicoplanin trough concentration (C_min_) > 10 mg/L. The overall clinical efficacy rate was 87.20%, and the bacteriological clearance rate was 82.61%. Adverse events included renal injury (8.80%) and hepatic injury (1.60%), with renal injury occurring more frequently in patients with higher C_min_ values. Bivariate analysis showed that teicoplanin trough concentrations were significantly associated with creatinine clearance (CrCL) and plasma albumin levels. Patients with severe renal impairment (CrCL <30 mL min^−1^) were more likely to achieve the target trough concentration. In addition, the incidence of renal injury increased significantly when the trough concentration exceeded 20 mg/L.

**Conclusion:**

A reliable, accurate, and reproducible HPLC method for the therapeutic drug monitoring of teicoplanin was successfully developed and validated. Application of the validated method presented substantial interindividual variability in teicoplanin plasma concentrations, with CrCL and plasma albumin identified as major factors associated with drug exposure. These findings provide a reliable analytical foundation for future studies of individualized teicoplanin dosing. However, given the retrospective observational design and the absence of real-time therapeutic drug monitoring-guided dose adjustment, the observed associations between trough concentrations and clinical outcomes should be interpreted as exploratory and hypothesis-generating rather than causal.

## 1 Introduction

Teicoplanin is a bactericidal glycopeptide antibiotic produced by *Actinomyces* species that exhibits potent activity against both aerobic and anaerobic Gram-positive bacteria. It is widely used as a first-line treatment for infections caused by Gram-positive cocci, including methicillin-resistant *Staphylococcus aureus* (MRSA) (Moñino-Dominguez et al., 2025). As a time-dependent antimicrobial agent, teicoplanin requires maintenance of an adequate plasma trough concentration (C_min_) to achieve optimal antibacterial activity (Matsumoto et al., 2016). Current guidelines recommend maintaining a C_min_ of at least 10 mg/L for mild to moderate infections and 15–30 mg/L for severe infections, including infective endocarditis (Hanai et al., 2022). Renal impairment reduces teicoplanin clearance (Zhou et al., 2022). Substantial pharmacokinetic (PK) variability has been reported among patients of advanced age and those with renal dysfunction, severe infections, or hypoproteinemia. These pathophysiological conditions contribute to marked interindividual and intraindividual variability in the distribution and elimination of teicoplanin (Shi et al., 2022; Chen et al., 2023). High-performance liquid chromatography (HPLC) has been widely used to determine plasma teicoplanin concentrations. Previously reported methods have used either the external standard method or internal standards such as piperacillin sodium, deuterated teicoplanin, or vancomycin (Wang and Yao, 2022; Meng et al., 2017; Hanada et al., 2005; Kim et al., 2016). However, these methods exhibit considerable variability in analytical performance, with reported linear ranges of 12.0–89.0 mg/L, 1.75–63.0 mg/L, and 1.0–100.0 mg/L, and some requiring analytical run times exceeding 30 min (Zhang et al., 2021).

In the present study, a rapid and practical HPLC method suitable for routine clinical application was developed for the determination of plasma teicoplanin concentrations. Factors associated with plasma teicoplanin concentrations were investigated, and their association with clinical efficacy were evaluated.

## 2 Data, participants, and methods

### 2.1 Research participants

A cohort of 125 adult patients who received teicoplanin for anti-infective treatment at the Second Hospital of Hebei Medical University between June 2022 and March 2023 were included in this study.

Inclusion criteria: (1) confirmed or suspected Gram-positive bacterial infection requiring teicoplanin therapy; (2) age ≥18 years; and (3) provision of informed consent to undergo plasma teicoplanin concentration monitoring during treatment.

Exclusion criteria: (1) pregnancy or lactation; (2) treatment duration <3 days; (3) absence of laboratory monitoring data for inflammatory markers prior to and following treatment; (4) topical administration of teicoplanin; and (5) receipt of continuous renal replacement therapy during treatment.

The study protocol was approved by the Ethics Committee of the Second Hospital of Hebei Medical University (No. 2022-R342). Written informed consent was obtained from all participants or their legally authorized representatives. For participants who were critically ill and unable to provide informed consent because of impaired consciousness or mechanical ventilation, written informed consent was obtained from their next of kin or legal guardians in accordance with the Declaration of Helsinki and the relevant Chinese regulations governing the ethical review of biomedical research involving human participants.

### 2.2 Dosing regimen

According to current clinical practice guidelines, a loading dose of teicoplanin is required because of its prolonged elimination half-life to achieve therapeutic plasma concentrations rapidly. For most Gram-positive bacterial infections, including pneumonia and skin and soft tissue infections, a loading dose of 400 mg every 12 h (q12h) is recommended, followed by a maintenance dose of 400 mg once (qd) after three loading doses (Tegas, 2017). For infective endocarditis and other severe infections, a loading dose of 800 mg q12h administered for three to five doses is recommended, followed by a maintenance dose of 800 mg qd.

According to the clinical practice guidelines for therapeutic drug monitoring (TDM) of teicoplanin jointly issued by the Japanese Society of Chemotherapy and the Japanese Society of Therapeutic Drug Monitoring, the initial TDM assessment should be performed after completion of the loading phase. The trough concentration should be measured immediately before administration of the third or fourth maintenance dose, which generally corresponds to Day 3 or Day 4 of treatment, depending on the dosing regimen used on the first day of therapy (Hanai et al., 2022).

### 2.3 Medications and instruments

Reference standards used for HPLC analysis included a teicoplanin reference standard (potency: 1 mg:892 U; batch No. 130374-202103; China Institute for Food and Drug Control) and quetiapine fumarate (purity: 99.6%; batch No. 100815-201904; China Institute for Food and Drug Control). The commercially available teicoplanin formulation (Targocid®; Sanofi; 400 mg/vial; batch No. H20160587) was administered to study participants. The vials were stored at 2 °C–8 °C until use.

The analytical instruments included an HPLC system (Waters, United States of America), an electronic analytical balance (Sartorius, Germany), a high-speed centrifuge (Abbott, United States of America), and a low-temperature refrigerator (Sanyo, Japan). Chromatographic separation was performed using a Waters Symmetry C18 column (4.6 mm × 250 mm, 5 μm).

### 2.4 Plasma concentration determination

#### 2.4.1 Chromatographic conditions

Chromatographic analysis was performed using a Waters Symmetry C18 column (4.6 mm × 250 mm, 5 μm particle size). The mobile phase consisted of acetonitrile and 0.01 mol/L sodium dihydrogen phosphate buffer at a ratio of 24:76 (v/v), with the pH adjusted to 3.29 using phosphoric acid. Prior to use, the mobile phase was filtered through a 0.45 μm membrane filter and degassed. The column temperature was maintained at 35 °C, the flow rate was 1.0 mL/min, and ultraviolet detection was performed at 240 nm. Quetiapine fumarate was used as the internal standard, and the injection volume was 20 μL. Prior to sample analysis, the HPLC system was equilibrated according to the standard operating procedures provided by the manufacturer. Upon completion of the analysis, the system was flushed with acetonitrile for 30 min.

#### 2.4.2 Preparation of solutions

A teicoplanin stock solution (1 mg/mL) was prepared by accurately weighing 10 mg of the teicoplanin reference standard, dissolving it in purified water, and adjusting the volume to 10 mL. The stock solution was serially diluted with purified water to obtain working solutions with concentrations of 1,000, 500, 250, 125, 62.5, 31.25, and 15.625 μg/mL. Both the stock and working solutions were stored at 4 °C until use. For preparation of the internal standard, 10 mg of quetiapine fumarate reference standard was dissolved in a 50% methanol–water solution, and the volume was adjusted to 10 mL to obtain a stock solution with a concentration of 1 mg/mL. A working internal standard solution (10 μg/mL) was prepared by diluting the stock solution with 50% methanol–water solution and was stored at 4 °C until use.

#### 2.4.3 Sample preparation

For plasma sample preparation, 200 μL of human blank plasma was mixed with 20 μL of the internal standard solution and vortex-mixed for 30 s. Proteins were precipitated by adding 600 μL of acetonitrile, followed by vortex mixing for 1 min. The mixture was then centrifuged at 1.2 × 10^4^ g for 10 min. Subsequently, 640 μL of the supernatant was collected, evaporated to dryness under a stream of nitrogen, and reconstituted with 200 μL of an acetonitrile–water mixture (6:19, v/v). After vortex mixing and centrifugation for 5 min, the resulting supernatant was collected and used for chromatographic analysis.

#### 2.4.4 Specificity

Blank human plasma from six different healthy donors, teicoplanin reference solutions, and plasma samples obtained from participants were processed according to the procedure described in Section 2.4.3 and analyzed under the chromatographic conditions described in Section 2.4.1.

#### 2.4.5 Calibration curve and lower limit of quantification

A series of simulated plasma samples was prepared by adding 20 μL of each teicoplanin working solution to separate 1.5 mL centrifuge tubes. The solutions were evaporated to dryness under a stream of nitrogen in a 50 °C water bath and subsequently mixed with 200 μL of blank plasma and 20 μL of the internal standard solution (10 μg/mL). Simulated plasma samples with final teicoplanin concentrations of 100, 50, 25, 12.5, 6.25, 3.125, and 1.5625 μg/mL were prepared, vortex-mixed for 30 s, and processed as described in Section 2.4.3. Linear regression analysis was performed using teicoplanin concentration as the independent variable (X) and the ratio of the teicoplanin peak area to the internal standard peak area as the dependent variable (Y). Weighted least-squares regression was used to construct the calibration curve. The lower limit of quantification (LLOQ) was defined as the lowest concentration corresponding to a signal-to-noise ratio of 10.

#### 2.4.6 Precision and recovery

Quality control (QC) samples at low (4 μg/mL), medium (25 μg/mL), and high (75 μg/mL) concentrations were independently prepared from separate stock solutions, distinct from those used for the calibration curve, and were processed and analyzed in each analytical run. For intra-day and inter-day precision and recovery assessment, five replicates of each QC concentration were prepared. The samples were processed as described in Section 2.4.3, and the peak areas of teicoplanin and the internal standard were determined. Absolute recovery was calculated as the ratio of the teicoplanin peak area obtained from the processed plasma samples to that obtained by direct injection of the corresponding standard solution. Relative recovery was defined as the ratio of the measured concentration to the theoretical concentration derived from the calibration curve. Intra-day and inter-day precision were assessed over three consecutive days.

#### 2.4.7 Stability

Simulated plasma samples containing teicoplanin at low, medium, and high concentrations (4, 25, and 75 μg/mL, respectively) were prepared in quintuplicate. Stability was assessed under the following storage conditions: storage at 4 °C for 24 h, three freeze–thaw cycles, room temperature for 12 h, and −40 °C for 7 days. Following each storage condition, the samples was processed according to the procedure described in Section 2.4.3 before analysis.

### 2.5 Observation indicators and evaluation criteria

#### 2.5.1 Observation indicators

Clinical data were extracted from the hospital’s electronic medical record system for all eligible participants. The collected variables included sex, age, body mass index (BMI), comorbidities, clinical symptoms, site of infection, body temperature, albumin (ALB), total protein, white blood cell count, procalcitonin, interleukin-6, C-reactive protein, neutrophil percentage, heparin-binding protein, serum creatinine (Scr), bacterial culture and antimicrobial susceptibility test results, loading dose, maintenance dose, and concomitant medications. Clinical and laboratory data were collected before and after teicoplanin administration.

#### 2.5.2 Evaluation of clinical efficacy

Clinical efficacy was assessed according to the *Technical Guidelines for Clinical Trials of Antimicrobial Drugs*, which classify therapeutic response into four categories (Writing Group of Antimicrobial Drug Clinical Trial Technology Guideline, 2012): Cured: Complete resolution of clinical symptoms and signs, normalization of laboratory findings, and eradication of the causative pathogen. Marked effect: Significant clinical improvement, with one of the following remaining abnormal: clinical symptoms, clinical signs, laboratory findings, or microbiological findings. Improved: Partial clinical improvement, with two or more of the above parameters remaining abnormal. Ineffective: No clinical improvement or clinical deterioration within 72 h after initiation of treatment. The clinical effective rate was calculated using the following formula:

Clinical effective rate (%) = (number of cured cases + number of cases with a marked effect)/total number of cases × 100%.

Bacteriological efficacy was classified into three categories: complete clearance, partial clearance, and no clearance. Complete clearance: Absence of the baseline pathogen in post-treatment cultures. Partial clearance: Reduction in the bacterial load of the baseline Gram-positive coccal pathogen compared with baseline. No clearance: Persistence of the baseline pathogen without a reduction in bacterial load. The bacterial clearance rate was calculated using the following formula: Bacterial clearance = (complete clearance + partial clearance)/(total Gram-positive cocci infections) × 100%. In this study, Gram-positive coccal pathogens included *S. aureus* (methicillin-resistant), *Staphylococcus* hominis, and *Staphylococcus* epidermidis.

#### 2.5.3 Safety evaluation

Adverse effects associated with teicoplanin included cutaneous and subcutaneous tissue disorders, hematologic abnormalities, and hepatic or renal injury (Atkar and Kapur, 2021). Renal injury was defined as kidney injury occurring during teicoplanin therapy or within 72 h after treatment discontinuation. Baseline renal function was determined using CrCL, calculated from the most recent Scr measurement obtained prior to treatment. Renal injury was diagnosed according to the 2012 *Kidney Disease: Improving Global Outcomes* (KDIGO) criteria and was defined by the presence of the following: an increase in Scr of ≥0.3 mg/dL (>26.5 μmol/L) within 48 h, an increase in Scr to ≥1.5 times the baseline value within 7 days, or a urine output of <0.5 mL/kg/h for ≥6 h. CrCL was classified as severe impairment (<30 mL/min), moderate impairment (30–60 mL/min), mild impairment (60–90 mL/min), normal function (90–120 mL/min), or hyperfiltration (>120 mL/min) (Kellum et al., 2013). Creatinine clearance was calculated using the Cockcroft–Gault equation: CrCL (mL/min) = [(140 - age) × weight (kg)]/[0.818 × Scr (µmol/L)] (result for females × 0.85).

Hepatic injury: Hepatic injury was defined as an alanine aminotransferase (ALT) level ≥3 times the upper limit of normal after treatment or ≥3 times the pre-treatment ALT value in participants with abnormal baseline ALT levels (Drug Induced Liver Disease Study Group and Chinese, 2015). Thrombocytopenia: Thrombocytopenia was defined as a platelet count of <100 × 10^9^/L after treatment (Song et al., 2020).

### 2.6 Statistical analysis

Statistical analyses were performed using SPSS version 25.0. Continuous variables with a normal distribution are presented as the mean ± standard deviation (SD) and were compared using the independent-samples *t*-test. Variables with a non-normal distribution are presented as the median (Q1, Q3) and were compared using the Mann–Whitney *U* test. Categorical variables are presented as frequencies and percentages and were compared using the chi-squared (χ^2^) test or Fisher’s exact test. Factors associated with plasma teicoplanin concentrations and clinical efficacy were initially assessed using univariate logistic regression analysis. Variables with *p* < 0.1 in the univariate analysis were entered into the multivariate logistic regression model after assessment for potential confounding and collinearity. A two-sided *p* value <0.05 was considered statistically significant.

## 3 Results

### 3.1 Methodological evaluation

#### 3.1.1 Specificity

Representative chromatograms of teicoplanin and the internal standard (quetiapine fumarate) are presented in Figure 1. The retention times were 14.46 min for the major teicoplanin component and 10.76 min for the internal standard. The multiple teicoplanin peaks corresponded to the naturally occurring A2-1 to A2-5 components and were consistent with the previously reported HPLC profiles. None of the six blank plasma lots from different healthy donors showed interfering peaks at the retention times of teicoplanin or the internal standard, confirming the selectivity of the method.

**FIGURE 1:**
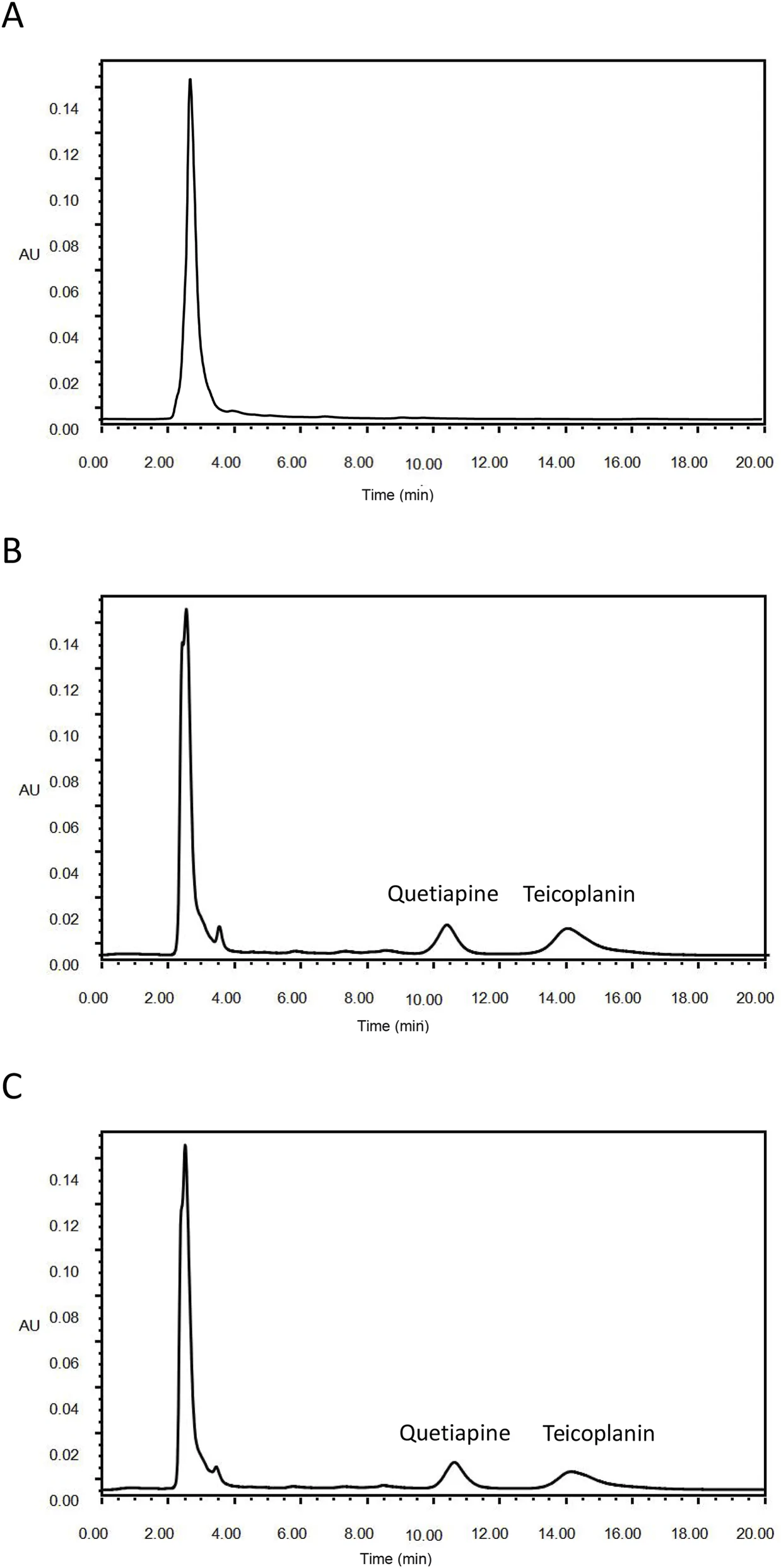
Representative high-performance liquid chromatograms of teicoplanin. **(A)** Blank human plasma. **(B)** Blank plasma spiked with teicoplanin and the internal standard (quetiapine fumarate). **(C)** Plasma sample obtained from a study participant receiving teicoplanin therapy. Quetiapine fumarate and the major teicoplanin component eluted at approximately 10.76 and 14.46 min, respectively. The cluster of teicoplanin-related peaks is consistent with the known multicomponent composition of teicoplanin, whose major A2 fraction comprises the A2-1 to A2-5 components (Jehl et al., 1988).

#### 3.1.2 Calibration curve and lower limit of quantification

Weighted least-squares regression (1/X^2^) demonstrated a linear relationship between the peak area ratio of teicoplanin to the internal standard (Y) and teicoplanin concentration (X) over the range of 1.5625–100 μg/mL (Figure 2), with the regression equation Y = 0.0171X + 0.0441 (*R* ^2^ = 0.9996). The LLOQ was 1.56 μg/mL.

**FIGURE 2:**
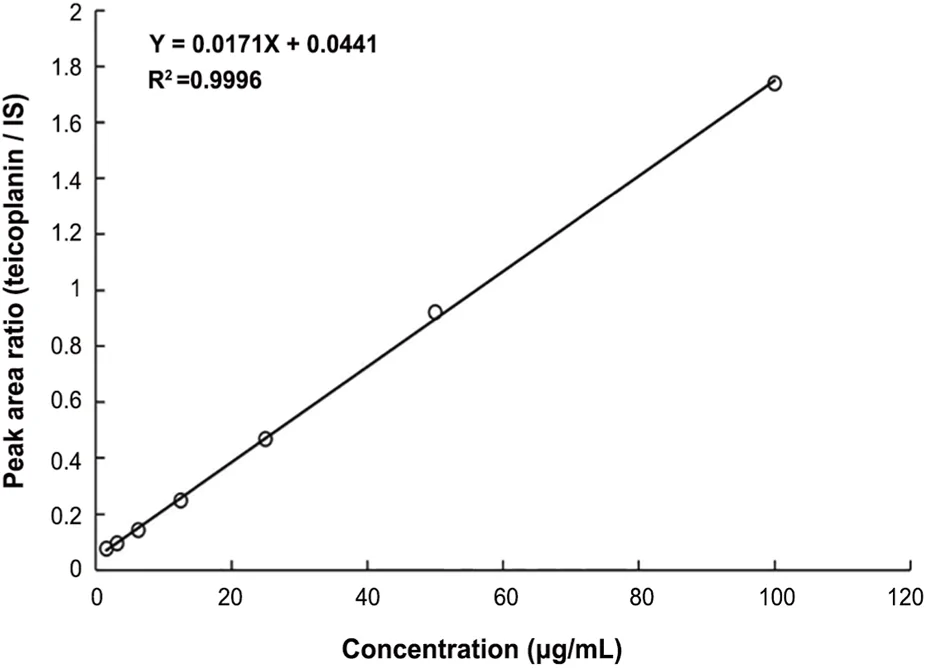
Representative calibration curve for teicoplanin determination by HPLC. Weighted linear regression (1/X^2^) over the concentration range of 1.5625–100 μg/mL yielded Y = 0.0171X + 0.0441 (R^2^ = 0.9996).

#### 3.1.3 Precision and recovery

The intra-day and inter-day relative standard deviations (RSDs) at the low-, medium-, and high-concentration quality control levels were all below 7.1%. Absolute and relative recovery values were within the predefined acceptable ranges (Table 1), indicating satisfactory precision and accuracy.

**TABLE 1:** Precision and recovery of the HPLC method for teicoplanin determination (mean ± SD, *n* = 5).

Concentration (μg/mL)	Intra-day measured concentration (μg/mL)	RSD (%)	Inter-day measured concentration (μg/mL)	RSD (%)	Absolute recovery (%)	Relative recovery (%)
4	3.78 ± 0.22	5.9	3.88 ± 0.27	7.1	108.7 ± 3.6	92.4 ± 6.3
25	25.47 ± 0.93	3.7	24.92 ± 1.09	4.4	94.8 ± 6.8	101.1 ± 5.1
75	74.51 ± 0.81	1.1	75.26 ± 2.25	3.0	89.4 ± 2.1	100.3 ± 2.4

#### 3.1.4 Stability

Teicoplanin remained stable after storage at 4 °C for 24 h, following three freeze–thaw cycles, after storage at room temperature for 12 h, and after storage at −40 °C for 7 days (Table 2).

**TABLE 2:** Stability of teicoplanin under different storage conditions (mean ± SD, n = 5).

Nominal concentration (μg/mL)	Room temperature, 12 h	RSD (%)	4 °C, 24 h	RSD (%)	Three freeze-thaw cycles	RSD (%)	Frozen storage, 21 days	RSD (%)
4	3.58 ± 0.15	4.1	3.46 ± 0.19	5.4	3.57 ± 0.21	5.8	3.60 ± 0.12	3.3
25	24.17 ± 0.71	2.9	23.09 ± 0.86	3.7	22.06 ± 0.73	3.3	21.66 ± 1.12	5.2
75	76.17 ± 3.50	4.6	75.97 ± 1.63	2.1	75.74 ± 3.79	5.0	76.05 ± 3.17	4.2

### 3.2 Baseline characteristics

A total of 125 participants were included in the analysis. Baseline demographic and clinical characteristics are summarized in Table 3. The study population comprised 68 males and 57 females, with an age range of 34–78 years, a mean age of 61.25 ± 9.20 years, a mean body weight of 70.5 ± 14.1 kg, and a mean BMI of 25.41 ± 3.57 kg/m^2^ (all values are presented as mean ± standard deviation, SD). All participants received a loading dose of 400 mg every 12 h for three doses, followed by a maintenance dose of 400 mg once daily. The mean treatment duration was 7.80 ± 2.76 days (mean ± SD). The primary diagnoses were lung infection (107 cases) and bloodstream infection (18 cases). Etiological testing was performed in 116 participants, among whom methicillin-resistant Gram-positive cocci were identified in 23 cases.

**TABLE 3:** Baseline characteristics of the study participants.

Characteristic	Value
Sex (M/F)	68/57
Age (years)	61.25 ± 9.20
Body weight (kg)	70.5 ± 14.1
Body mass index (kg/m^2^)	25.41 ± 3.57
Duration of teicoplanin therapy (days)	7.80 ± 2.76
Site of infection (n = 125)	
Lung	107
Bloodstream	18
Methicillin-resistant Gram-positive isolates (n = 23)	
*Staphylococcus haemolyticus*	4
*Staphylococcus hominis*	7
*Staphylococcus aureus*	12

Data are presented as mean ± SD, for normally distributed continuous variables (age, weight, BMI, and treatment duration).

### 3.3 Plasma teicoplanin concentrations

Plasma trough teicoplanin concentrations (C_min_) ranged from 1.6 to 39.7 μg/mL, with a mean concentration of 11.6 ± 10.0 μg/mL (mean ± SD). The overall target attainment rate for C_min_ > 10 μg/mL was 45.6% (57/125) (Table 4).

**TABLE 4:** Association of baseline characteristics with target attainment of teicoplanin trough concentrations.

Characteristic	Participants achieving target (*n*)	Target attainment (%)	χ^2^	*P value*
Sex				
Male	30/68	44.12	0.132	0.716
Female	27/57	47.37		
Age (years)				
<55	11/25	44.00	1.081	0.941
55–64	23/52	44.23		
65–75	23/47	48.94		
>75	0/1	0		
Body weight (kg)				
<60	12/19	63.16	6.850	0.033
60–80	35/71	49.30		
>80	10/35	28.57		
BMI (kg/m^2^)				
<18.5	2/5	40.00	1.493	0.684
18.5–23.9	24/55	43.64		
>24.0	31/65	47.69		
CrCL (mL/min)				
<30	2/2	100.00	18.970	0.000
30–60	26/39	66.67		
60–90	22/50	44.00		
90–120	6/22	27.27		
>120	1/12	8.33		
ALB (g/L)				
<35	26/73	35.62	7.051	0.008
≥35	31/52	59.62		

### 3.4 Factors associated with plasma teicoplanin concentration

Univariate analysis was performed to evaluate factors associated with attainment of the target trough concentration (C_min_ > 10 μg/mL), including sex, age, body weight, BMI, CrCL, and serum albumin (ALB) (Table 4). Body weight (χ^2^ = 6.850, *p* = 0.033), CrCL (χ^2^ = 18.970, *p* < 0.001), and ALB (χ^2^ = 7.051, *p* = 0.008) were significantly associated with target concentration attainment, whereas sex, age, and BMI were not (*p* > 0.05 for all).

Multivariable logistic regression analysis identified body weight, CrCL, and ALB as independent factors associated with teicoplanin trough concentrations (Table 4). No significant associations were observed for sex, age, or BMI (*p* > 0.05).

### 3.5 Clinical efficacy

#### 3.5.1 Clinical outcomes

The overall clinical effectiveness rate was 87.20% (109/125). The clinical effectiveness rates by C_min_ subgroup are presented in Table 5. Although the clinical effectiveness rate showed an increasing trend with higher C_min_ (85.29%, 85.71%, and 95.45% for C_min_ < 10, 10–20, and >20 μg/mL, respectively), the difference was not statistically significant (χ^2^ = 2.008, *p* = 0.366).

**TABLE 5:** Clinical effectiveness of teicoplanin by C_min_ subgroup.

C_min_ (μg/mL)	Participants (n)	Clinically effective (n)	Clinical effectiveness (%)	χ^2^	*P* value
<10	68	58	85.29	2.008	0.366
10–20	35	30	85.71		
>20	22	21	95.45		

Bacteriological clearance was assessed among the 23 patients with confirmed methicillin-resistant Gram-positive coccal infections. None of these patients received other concomitant anti-Gram-positive antibiotics (e.g., vancomycin, linezolid, or daptomycin) during teicoplanin therapy. The clearance rates by C_min_ subgroup are presented in Table 6. The bacteriological clearance rates showed an increasing trend with higher C_min_ (60.00%, 81.82%, and 100.00%, respectively), but the difference was not statistically significant (χ^2^ = 1.694, *p* = 0.379).

**TABLE 6:** Bacteriological clearance rate of teicoplanin by C_min_ subgroup among patients with confirmed methicillin-resistant Gram-positive coccal infections.

C_min_ (μg/mL)	Participants with positive cultures (n)	Bacteriological clearance (n)	Bacteriological clearance rate (%)	χ^2^	*p* value
<10	10	6	60.00	1.694	0.379
10–20	11	9	81.82		
>20	2	2	100.00		

#### 3.5.2 Factors associated with clinical effectiveness

Binary logistic regression analysis demonstrated that hypoproteinemia, hepatic dysfunction, and impaired CrCL were significantly associated with reduced clinical effectiveness (*p* < 0.05) (Table 7).

**TABLE 7:** Association of baseline clinical characteristics with clinical effectiveness.

Characteristic	Participants (n)	Clinically effective (n)	Clinical effectiveness (%)	χ^2^	*p* value
Age (years)					
<65	77	65	84.4%	1.393	0.238
≥65	48	44	91.7%		
Duration of teicoplanin therapy (d)					
<7	56	47	83.9%	0.973	0.324
≥7	69	62	89.8%		
Renal impairment					
Yes	40	30	75.0%	7.844	0.005
No	85	79	92.9%		
Hepatic injury					
Yes	11	7	63.6%	3.9081	0.048
No	114	102	89.5%		
Hypoalbuminemia					
Yes	73	59	80.8%	4.647	0.031
No	52	49	94.2%		

### 3.6 Safety

No cutaneous or hematological adverse events were observed during teicoplanin therapy. Renal injury occurred in 11 participants (8.80%), whereas hepatic injury occurred in two participants (1.60%). The incidence of renal injury increased across higher C_min_ categories (Table 8). However, given the retrospective observational design and the absence of adjustment for concomitant nephrotoxic medications, this finding should be interpreted as a descriptive association rather than evidence of a causal relationship between elevated teicoplanin concentrations and renal injury.

**TABLE 8:** Incidence of renal and hepatic injury according to teicoplanin trough concentration.

Teicoplanin trough concentration (μg/mL)	Participants (n)	Renal injury (n)	Incidence (%)	Hepatic injury (n)	Incidence (%)
<10	68	4	5.88%	0	0.00%
10–20	35	3	8.57%	2	5.71%
>20	22	4	18.18%	0	0.00%

## 4 Discussion

The HPLC method developed in this study demonstrated high sensitivity, rapid analysis, and operational simplicity, making it suitable for routine TDM of teicoplanin in clinical practice. Previous HPLC methods have commonly used the external standard method or internal standards such as piperacillin sodium, deuterated teicoplanin, or vancomycin. In the present study, quetiapine fumarate was selected as the internal standard. Compared with the method reported by Sun et al., which used nor vancomycin as the internal standard, quetiapine fumarate is less expensive, readily available, and provides favorable chromatographic separation (Memiş et al., 2025). Compared with the method described by Nakano et al., which employed a teicoplanin analog as the internal standard, the internal standard used in the present method is more economical and practical, making it more suitable for routine clinical testing (Lee et al., 2021).

Although piperacillin sodium has frequently been used as an internal standard for teicoplanin assays, it has several limitations. First, concomitant administration of teicoplanin with piperacillin/tazobactam or piperacillin/sulbactam may produce interfering internal standard peaks, potentially compromising quantitive accuracy. Second, the limited solution stability of piperacillin sodium may adversely affect the assay reproducibility and reliability (Meng et al., 2017; Bassetti et al., 2018; Jenkins et al., 2022). In contrast, the external standard method is more susceptible to analytical variability because identical chromatographic conditions are difficult to maintain across multiple analytical runs (Snyder et al., 2010).

The optimized chromatographic conditions established in this study enabled rapid separation, with a retention time of 14.656 min for teicoplanin. By comparison, the method reported by Pea et al. required an analytical run time exceeding 25 min (Pea et al., 2003). An acetonitrile–phosphate buffer mobile phase (pH 3.29) was selected. Compared with the trifluoroacetic acid–acetonitrile mobile phase reported by Wong et al., this system is expected to reduce potential chromatographic column deterioration associated with strongly acidic additives, thereby extending column service life (Wong et al., 2013). The mobile phase composition was further optimized according to the relevant literature and repeated experimental adjustments. The final acetonitrile-to-0.01 mol/L sodium dihydrogen phosphate buffer ratio of 24:76 (v/v) achieved satisfactory separation of teicoplanin and quetiapine fumarate with acceptable peak symmetry.

The LLOQ was 1.5625 μg/mL, and the assay was linear over the concentration range of 1.5625–100 μg/mL. This represents an improvement over the HPLC method reported by Matsumoto et al., which had an LLOQ of 3.0 μg/mL (Matsumoto et al., 2016). Compared with the method described by Nakano et al. (linear range, 5–100 μg/mL), this method enables more accurate quantification of low-concentration samples, thereby better meeting the clinical needs of patients receiving low-dose therapy (Lee et al., 2021). Simple pretreatment was performed using a simple protein precipitation procedure. Compared with the solid-phase extraction method reported by Liu et al., this approach reduces both sample processing time and cost (Lee et al., 2021). Processing a single sample requires approximately 30 min, whereas solid-phase extraction typically requires more than 45 min. These characteristics improve the feasibility of routine implementation in clinical laboratories. Considering reagent costs, equipment maintenance, and labor requirements, the per-sample analytical cost of this method is approximately 60% of that reported by (Chen et al. (2023)). The HPLC system used in this study is widely available in clinical laboratories and does not require mass spectrometry equipment, further supporting its practicality and feasibility for routine clinical use.

Despite these methodological advantages, two limitations regarding the internal standard should be acknowledged. First, although quetiapine fumarate was selected because of its favorable chromatographic characteristics, cost-effectiveness, and ready availability, its stability in plasma under the specific experimental conditions used in this study, including room temperature exposure, protein precipitation with acetonitrile, nitrogen evaporation, and sample reconstitution, was not assessed independently. Nevertheless, quetiapine fumarate is a certified reference material (purity, 99.6%) with well-established chemical stability. In addition, the consistently low variability of the internal standard peak area (RSD <5% across all analytical runs) together with the excellent relative recovery of teicoplanin (89.41%–108.70%) provides indirect evidence supporting the stability and suitability of quetiapine fumarate under the analytical conditions employed.

Second, potential chromatographic interference from a broad range of concomitantly administered medications was not evaluated systematically. This limitation is particularly relevant because piperacillin has previously been used as an internal standard for teicoplanin assays. Nevertheless, a retrospective review of chromatograms from participants who received concomitant piperacillin/tazobactam demonstrated no interfering peaks at the retention time of quetiapine fumarate (10.759 min). Published HPLC studies have shown that piperacillin and tazobactam elute at approximately 4–6 min and 3–4 min, respectively, under similar reversed-phase chromatographic conditions, substantially earlier than quetiapine fumarate (Veillette et al., 2016; Legrand et al., 2008). Although these observations support the specificity of the present method, a prospective evaluation of potential interference from a broader panel of commonly co-administered medications would further strengthen its analytical validation.

Teicoplanin has a prolonged elimination half-life of approximately 70–100 h. Under the standard clinical dosing regimen (e.g., 400 mg every 12 h on Day 1, followed by 400 mg once daily), patients typically receive two or three loading doses on the first day of treatment. Given its prolonged elimination half-life, following once-daily maintenance dosing on Days 2 and 3, the trough concentration measured on the morning of Day 4 (i.e., before the fourth maintenance dose) is expected to approach steady state and provides a clinically appropriate time point for TDM and subsequent dose adjustment. Ramos-Martin et al. noted in a review that trough concentrations should be measured at steady state, usually after the third or fourth dose (Ogawa et al., 2013). Similarly, Matthews et al. reported in a systematic review that the initial trough concentration is commonly measured after completion of the loading phase, immediately before administration of the fourth dose (Mouton et al., 2025). Accordingly, in the present study, the first blood sample for teicoplanin TDM was collected 30 min before dosing on Day 4.

Current guidelines recommend maintaining teicoplanin trough concentrations (C_min_) ≥ 10 μg/mL for the treatment of common Gram-positive coccal infections (Hanai et al., 2022). Analysis of TDM data from 125 participants showed that the target attainment rate under the standard teicoplanin dosing regimen was relatively low (45.6%). Body weight, CrCL, and ALB levels were identified as independent factors associated with trough concentrations.

CrCL was the factor most strongly associated with teicoplanin trough concentrations and demonstrated a significant negative correlation inverse association with C_min_. This finding is consistent with the established pharmacokinetic characteristics of teicoplanin, which is primarily excreted unchanged by the kidneys (approximately 60%–70%) (Tegas, 2017). As renal function declines, teicoplanin clearance decreases, resulting in drug accumulation and higher trough concentrations. This pharmacokinetic relationship forms the basis for adjusting teicoplanin dosing according to renal function in routine clinical practice. In critically ill individuals, an initial loading dose of 8–12 mg/kg has been reported to achieve target C_min_ more rapidly, with higher target attainment rates in those with a glomerular filtration rate (GFR) ≥ 60 mL/min. However, augmented renal clearance (CrCL ≥ 130 mL/min) may significantly reduce plasma concentrations of renally excreted drugs, including teicoplanin, potentially compromising therapeutic efficacy (Chen and Nicolau, 2020). Consistent with these findings, patients in the present study with moderate or severe renal insufficiency were more likely to achieve the target C_min_, whereas those with mild renal insufficiency, normal CrCL, or hyperfiltration had lower target attainment rates. Because relatively few participants had moderate or severe renal impairment, the overall proportion achieving C_min_ ≥ 10 μg/mL remained low, consistent with previous reports (Barbot et al., 2003).

Plasma ALB concentration was also identified as an independent factor associated with teicoplanin trough concentrations. Participants with hypoalbuminemia (ALB <35 g/L) had a markedly lower target attainment rate than those with normal albumin concentrations. Because teicoplanin is highly protein-bound (>90%), reduced albumin concentrations increase the unbound fraction of the drug. Although a higher unbound fraction may theoretically enhance antibacterial activity, it may also increase drug clearance and alter the volume of distribution, ultimately reducing the total trough concentration (Ulldem et al., 2011). Among critically ill individuals, plasma ALB concentration has been reported to be significantly associated with teicoplanin clearance (Barbot et al., 2003).

The unbound fraction of teicoplanin varies considerably among individuals with hypoalbuminemia. In patients with severe hypoalbuminemia (median ALB, 1.6 g/dL), the unbound fraction ranged from 8% to 42%, whereas in those with moderate hypoalbuminemia (median ALB, 2.9 g/dL), it ranged from 3.4% to 18.8% (Mimoz et al., 2006). In general, a higher unbound fraction is associated with increased drug clearance. Multivariable analyses have identified severe hypoalbuminemia (ALB <2.2 g/dL) as an independent risk factor for reduced C_min_ (Yoshida et al., 2019). In the present study, 58.4% (73/125) of participants had hypoalbuminemia, and only 35.5% achieved C_min_ ≥ 10 μg/mL, approximately half the proportion observed among participants with normal ALB levels. These findings support the importance of TDM in individuals with hypoalbuminemia and suggest that dose adjustment may be necessary to achieve adequate total drug exposure in this population.

An important consideration in teicoplanin TDM is whether target attainment should be based on total or unbound drug concentrations. Teicoplanin is highly protein bound (approximately 90%), and only the unbound fraction is pharmacologically active. In individuals with hypoalbuminemia, reduced protein binding increases the unbound fraction, which may result in increased drug clearance and consequently lower total plasma concentrations while maintaining unbound concentrations within the therapeutic range. Conversely, elevated unbound trough concentrations have been associated with an increased risk of renal injury. Currently, total trough concentrations remain the standard parameter for routine clinical TDM because assays for unbound teicoplanin concentrations are not widely available. In individuals with severe hypoalbuminemia, measurement or estimation of unbound concentrations (e.g., using equilibrium protein-binding equations) may provide a more accurate basis for dose individualization. Further studies are warranted to evaluate the clinical utility of this approach.

Higher body weight, particularly >80 kg, was identified as an independent factor associated with subtherapeutic teicoplanin trough concentrations. This finding proposes that fixed-dose regimens may result in a relatively larger volume of distribution in individuals with higher body weight, thereby reducing systemic drug exposure and decreasing the likelihood of achieving therapeutic trough concentrations. These findings support the clinical use of individualized, weight-based dosing.

Population pharmacokinetic modeling remains the gold standard for characterizing interindividual variability in drug disposition. Most published population pharmacokinetic models have consistently identified CrCL and body weight as the principal covariates influencing teicoplanin clearance and volume of distribution (Ogawa et al., 2013; Broeker et al., 2019; Li et al., 2025). For example, the model developed by Pea et al. identified CrCL as the primary determinant of clearance, whereas body weight was significantly associated with the volume of the central compartment (Pea et al., 2003).

The present findings, in which impaired CrCL was associated with higher trough concentrations and higher body weight was associated with lower trough concentrations, are consistent with these established pharmacokinetic relationships and provide supporting evidence from a real-world clinical cohort. Although ALB has not been incorporated consistently into population pharmacokinetic models as CrCL or body weight, several more comprehensive models have identified albumin as a relevant covariate affecting protein binding and, consequently, drug clearance, consistent with the present findings (Roberts et al., 2013). However, several published models suggest that, in individuals with obesity, lean body weight or ideal body weight may predict the volume of distribution more accurately than actual body weight (Han et al., 2007).

In the present study, only actual body weight was assessed. Future studies should examine the relationships between different body weight metrics and target attainment to help refine individualized dosing strategies. Several advanced population pharmacokinetic models have identified other clinically relevant covariates, including concomitant medications, particularly nephrotoxic agents, and inflammatory markers such as C-reactive protein, which were not included in the present analysis (Mouton et al., 2026). These unmeasured variables may partly explain the substantial interindividual variability in teicoplanin concentrations observed among participants with similar renal function and body weight.

Teicoplanin is a time-dependent antimicrobial agent, and its clinical efficacy is theoretically related to maintenance of adequate trough serum concentrations (C_min_). In the present study, although both clinical effectiveness and bacteriological clearance tended to increase with increasing C_min_, the differences among the concentration subgroups were not statistically significant (Table 5 and 6). Given the retrospective observational design, these findings should be interpreted with caution.

Therapeutic drug concentrations were not used to guide real-time dose adjustments, and the absence of a statistically significant association between higher C_min_ and improved outcomes may reflect confounding by indication. Specifically, participants with more severe infections may have received higher doses or longer treatment durations, thereby obscuring a true exposure–response relationship. The non-significant *p* values presented in Table 5 should not be interpreted as evidence against a causal relationship between teicoplanin exposure and clinical efficacy.

Several additional factors may also have contributed to these findings. First, a substantial proportion of participants were critically ill and had multiple comorbidities, poor nutritional status, impaired immune function, and an unfavorable prognosis. Second, although microbiological testing was performed in 116 patients, only 23 had confirmed Gram-positive coccal infections. Teicoplanin was administered as targeted therapy in these patients, whereas most of the remaining patients received empirical antimicrobial therapy, potentially for infections caused by Gram-negative bacilli. Consequently, the observed clinical improvement may have reflected the effects of concomitant antimicrobial therapy rather than those of teicoplanin alone. Third, although a C_min_ ≥ 10 μg/mL may be recommended as the therapeutic target, this threshold primarily serves as an early pharmacokinetic marker and may not fully predict final clinical outcomes. Experimental and clinical evidence suggests that pharmacokinetic/pharmacodynamic indices, such as the area under the concentration–time curve to minimum inhibitory concentration ratio may better predict anti-MRSA activity than trough concentrations alone (Byrne et al., 2017; Ramos-Martin et al., 2017).

Notably, all 23 patients with confirmed MR-GPC infections received teicoplanin as the sole anti-Gram-positive agent, providing a relatively clean basis for evaluating the exposure–response relationship. In this subgroup, the bacteriological clearance rate showed a consistent increasing trend with higher Cmin, supporting the clinical relevance of achieving target trough concentrations, although statistical significance was not reached—possibly due to the limited sample size.

Prospective interventional studies incorporating TDM-guided dose adjustment are needed to determine if achieving C_min_ ≥ 10 μg/mL is causally associated with improved clinical outcomes. Further studies with larger cohorts are ensured to validate these findings.

Adverse reactions associated with teicoplanin, including drug-induced fever, rash, hepatic injury, renal injury, and thrombocytopenia, have been reported to be associated with plasma drug concentrations, particularly during high-dose or prolonged therapy (Atkar and Kapur, 2021; Pea, 2020). In the present study, renal injury was the most frequently observed adverse event (8.80%), followed by hepatic injury (1.60%). Notably, the incidence of renal injury increased across higher C_min_ categories, suggesting an association between elevated teicoplanin trough concentrations and renal injury. These findings underscore the importance of TDM and careful assessment of adverse events to support the safe and effective use of teicoplanin.

However, the observed association between higher teicoplanin trough concentrations and an increased incidence of renal injury should be interpreted with caution. Patients with higher C_min_ values in this cohort were more likely to be critically ill and, consequently, may have received multiple concomitant medications with nephrotoxic potential, including vancomycin, aminoglycosides, loop diuretics, iodinated contrast media, angiotensin-converting enzyme inhibitors, angiotensin II receptor blockers, and non-steroidal anti-inflammatory drugs, which were not systematically recorded or adjusted for in the present analysis.

Greater illness severity may itself have predisposed these patients to acute kidney injury through hemodynamic instability, sepsis-associated acute kidney injury, and other non-drug-related mechanisms (Hoste et al., 2015; Bellomo et al., 2017). Therefore, the higher incidence of renal injury observed among participants in the C_min_ > 20 μg/mL group should be regarded as a descriptive association and may reflect confounding by indication and polypharmacy rather than a direct nephrotoxic effect of teicoplanin. This contrasts with the more firmly established concentration-dependent nephrotoxicity of vancomycin, for which evidence is supported by prospective studies that adjusted for relevant confounding factors (Rybak et al., 2020). For teicoplanin, the causal relationship between elevated trough concentrations and nephrotoxicity remains to be established in well-designed prospective studies with adequate adjustment for concomitant nephrotoxic exposures.

Beyond the potential nephrotoxic effects of teicoplanin, it is important to recognize that patients in this cohort, particularly those with severe infections, were exposed to a complex pathophysiological conditions that independently predispose to both renal and cardiovascular injury. Systemic inflammation, sepsis-associated hemodynamic instability, and endothelial dysfunction are well-established contributors to acute kidney injury and myocardial dysfunction in critically ill individuals. Recent studies have further elucidated molecular mechanisms involved in cardiovascular remodeling and repair under conditions of inflammatory and hemodynamic stress. For example, Zhong et al. identified Mas-related G-protein-coupled receptor member D (MrgD), a novel component of the renin–angiotensin system, as an important modulator of pulmonary vascular remodeling and a potential therapeutic target for pulmonary hypertension (Zhong et al., 2025). Emerging evidence further suggests that non-canonical renin–angiotensin system pathways, including the Alamandine/MrgD axis, may suppress vascular calcification by inhibiting ferroptosis (Yao et al., 2026).

These mechanistic findings emphasize that cardiovascular and renal injury in individuals with severe infection is multifactorial and reflects complex interactions among drug exposure, systemic inflammation, and vascular homeostatic pathways. The higher incidence of renal injury observed among participants with C_min_ > 20 μg/mL should be interpreted within this broader pathophysiological context rather than attributed solely to teicoplanin exposure.

This study has several limitations. First, because of the retrospective observational design, residual confounding could not be eliminated. For example, concomitant medications, particularly other nephrotoxic agents or drugs influencing protein binding may have affected both teicoplanin pharmacokinetics and the risk of renal injury. Second, key covariates, including ALB and CrCL, were not always measured at the same time as plasma teicoplanin concentrations. In critically ill individuals, these parameters may change rapidly, and this temporal mismatch may have reduced the precision of the analyses.

Third, the measured teicoplanin concentrations were not used to guide real-time dose adjustment. Consequently, the absence of a significant association between C_min_ and clinical efficacy (Table 5) should not be interpreted as evidence against a true exposure–response relationship. Rather, this finding may reflect confounding by indication, whereby patients with more severe infections received higher doses or longer treatment courses, biasing the observed associations toward the null. Therefore, this study primarily identifies factors associated with achieved through concentrations rather than demonstrating that prospective TDM-guided dose adjustment based on these factors improves clinical efficacy or safety. Prospective interventional studies are needed to determine the clinical utility of these covariates for TDM-guided dose optimization and to establish causal relationships between teicoplanin exposure and clinical outcomes. Fourth, concomitant administration of other nephrotoxic agents was not systematically recorded or adjusted for in the safety analysis, despite representing an important source of potential confounding. Patients with higher teicoplanin trough concentrations were more likely to have severe illness and to receive multiple nephrotoxic medications. Therefore, the higher incidence of renal injury observed in the high-concentration group may have been partially or entirely attributable to these unmeasured confounders rather than to teicoplanin exposure alone. This limitation substantially restricts the causal interpretation of the safety findings.

Fifth, although the stability and recovery of teicoplanin were validated under multiple experimental conditions (Table 2), the stability of the internal standard (quetiapine fumarate) in plasma was not assessed independently. However, its established chemical stability and the consistent internal standard peak areas observed across analytical runs support its suitability under the experimental conditions used in this study. Direct assessment of internal standard stability under these conditions would further strengthen the analytical method. In addition, potential chromatographic interference from a comprehensive range of concomitantly administered medications was not assessed systematically. Although no interfering peaks were observed at the retention time of the internal standard in chromatograms from participants who received concomitant piperacillin/tazobactam, a comprehensive interference study would provide more robust evidence supporting the specificity of the analytical method.

## 5 Conclusion

Teicoplanin demonstrated favorable clinical effectiveness in the treatment of Gram-positive bacterial infections, with a relatively low incidence of adverse events. Under the conventional dosing regimen evaluated in this study, the target attainment rate for trough concentrations was suboptimal. CrCL, ALB concentration, and body weight were identified as independent factors associated with teicoplanin trough concentrations, providing further insight into the sources of interindividual pharmacokinetic variability. These variables should be considered as potential covariates in future TDM-guided individualized dosing strategies. However, due to the retrospective observational design and the absence of TDM-guided dose adjustment, the observed associations between trough concentrations and clinical outcomes should be regarded as exploratory.

Prospective treatment interventional studies with larger sample sizes are warranted to determine whether incorporation of these patient-specific factors into TDM-guided dose optimization improves clinical outcomes and to establish the causal nature of the exposure–response relationship.

## Data Availability

The original contributions presented in the study are included in the article/supplementary material, further inquiries can be directed to the corresponding authors.
